# Effect of dilutional anemia that can be treated with only one unit of red blood cell transfusion on tissue oxygenation in cardiac surgery patients

**DOI:** 10.3906/sag-1901-213

**Published:** 2019-08-08

**Authors:** Büşra TEZCAN, Demet BÖLÜKBAŞI, Alev ŞAYLAN, Sema TURAN, Sultan Sevim YAKIN, Dilek KAZANCI, Ayşegül ÖZGÖK, Hija YAZICIOĞLU

**Affiliations:** 1 Department of Intensive Care, Ministry of Health Ankara City Hospital, Ankara Turkey; 2 Department of Anesthesiology and Reanimation, İstanbul Lütfi Kırdar Education and Research Hospital, İstanbul Turkey; 3 Department of Anesthesiology and Reanimation, Ministry of Health Ankara City Hospital, Ankara Turkey

**Keywords:** Cardiac surgical procedures, blood transfusion, microcirculation, lactate, arteriovenous carbon dioxide difference

## Abstract

**Background/aim:**

Cardiac surgery, especially in the presence of cardiopulmonary bypass (CPB), is associated with an inflammatory reaction that may promote microcirculatory alterations, in addition to the general impact on system hemodynamics. Anemia and transfusion make patients more susceptible to the deleterious effects of CPB. In this study, it was aimed to evaluate the effect of dilutional anemia, which is caused by CPB and can be treated with 1–2 units of red blood cell (RBC) transfusion, on global tissue oxygenation parameters in cardiac surgery patients.

**Materials and methods:**

This prospective observational study comprised 127 patients who had a relatively stable operation period without any major anesthetic or surgical complications (e.g., operation duration >5 h, bleeding or hemodilution requiring more than 1–2 units of RBCs, or unstable hemodynamics, requiring inotropic support of more than 5 µg/kg/min dopamine). Patients were observationally divided into two groups: minimally transfused (Group Tr) and nontransfused (Group NTr). Global tissue oxygenation parameters were evaluated after anesthesia induction (T1) and at the end of the operation (T3) and compared between the groups.

**Results:**

Group Tr consisted of patients who had significantly lower preoperative hemoglobin values than Group NTr patients. The dilutional anemia of all Group Tr patients could be corrected with 1 unit of RBCs. The lactate levels at T3, increment rates of lactate, and venoarterial carbon dioxide pressure difference (ΔpCO2) levels [(T3 – T1) : T1] in Group Tr were significantly higher than those in Group NTr.

**Conclusion:**

Dilutional anemia as a result of CPB mostly occurs in patients with borderline preoperative hemoglobin concentrations and its correction with RBC transfusion does not normalize the degree of microcirculatory and oxygenation problems, which the patients are already prone to because of the nature of CPB. Preventing dilutional anemia and transfusion, especially in patients with preoperative borderline hemoglobin levels, may therefore reduce the burden of impaired microcirculation-associated organ failure in on-pump cardiac surgery.

## 1. Introduction

Maintenance of microcirculatory homeostasis is essential for the preservation of organ function [1]. Cardiac surgery is associated with an inflammatory reaction that may promote alterations in the microcirculatory level, in addition to the general impact on hemodynamic status [2]. The use of cardiopulmonary bypass (CPB) additionally causes a broad range of changes in microcirculatory perfusion and oxygenation [1].

Anemia and transfusion not only make patients more prone to the deleterious effects of CPB but also aggravate the inflammatory response, oxidative stress, and renal hypoxia [3,4]. An intraoperative transfusion of red blood cells (RBCs) increases the risk of mortality and several morbidities. These risks are substantial for even one unit in general surgery patients [5].

The aim of this study was to compare tissue oxygenation, which was evaluated by blood lactate concentration, central venous oxygen saturation (ScvO2), and venoarterial carbon dioxide pressure difference (ΔpCO2), between patients who had dilutional anemia resulting from CPB and could be treated with a minimal RBC transfusion (1–2 units) and the patients who did not. Our hypothesis was that a minimal transfusion would lead to normalized global tissue oxygenation parameters that were supposed to be impaired by dilutional anemia resulting from CPB. 

## 2. Materials and methods

### 2.1. Patient sample and definitions

We designed and conducted a single-center, prospective, nonrandomized study that was approved by the Numune Education and Research Hospital’s Human Research Ethics Committee. The study was registered with ClinicalTrials.gov (NCT03245502). 

Patients scheduled for elective on-pump coronary artery bypass grafting (CABG) and valve replacement surgery were eligible to be included. Eligible patients gave prior informed written consent. The exclusion criteria were being <18 or >75 years of age, previous cardiac surgery, American Society of Anesthesiologists physical status of III–IV, preoperative ejection fraction of <45%, preexisting renal disease (serum creatinine >1.2 mg/dL), history of myocardial infarction, smoking, any coagulopathy or ongoing anticoagulation therapy, and preoperative anemia defined as hemoglobin of <12.0 g/dL in women and 13.0 g/dL in men. Patients with comorbid diseases, except for well-controlled diabetes mellitus and hypertension, were also excluded. Patients who had a relatively stable operation period without any major anesthetic or surgical complications (e.g., operation duration >5 h, bleeding or hemodilution requiring more than 2 units of RBCs, unstable hemodynamics requiring inotropic support of more than 5 µg/kg/min dopamine) were included in this observational study and were divided into 2 groups: transfused (Group Tr) and nontransfused (Group NTr). Group Tr patients were patients who had been transfused with 1–2 units of RBCs for the correction of dilutional anemia resulting from CPB. Blood transfusions were driven primarily by hemoglobin levels (7.0 g/dL) obtained from the blood gas analysis at the fifth minute of CPB.

To reduce the confounding effects of excessive blood loss, patients who received more than 2 units of erythrocytes were excluded from the study. The duration of storage of the RBCs was less than 10 days.

### 2.2. Anesthesia and surgery management

All surgeries were performed under general anesthesia with CPB by the same team of surgeons and anesthesiologists. Each patient’s radial artery was cannulated for blood sampling and invasive blood pressure monitorization. Next, anesthesia induction was performed via intravenous 1 mg/kg lidocaine, 0.1 mg/kg midazolam, 10–15 µg/kg fentanyl, and 0.5–0.7 mg/kg rocuronium. Following endotracheal intubation, the right internal jugular vein was also cannulated for blood sampling and central venous pressure monitorization. Anesthesia was maintained with intermittent fentanyl-midazolam bolus injections. Rocuronium was used as a neuromuscular blocker agent when needed. A mean perfusion pressure of 50 to 70 mmHg and CPB flow rates of 3.5 to 4 L min/m2 were maintained. Colloid solutions were not administered. 

### 2.3. Data collection

The following demographic and perioperative variables were collected for each patient: age (years), sex, body surface area (m2), preoperative hemoglobin levels (g/dL), type of surgery, operation, CPB and aortic cross-clamp durations (min), and total crystalloid administration (mL/kg). Before skin incision (T1) and after skin closure (T3), a standard arterial and central venous blood gas analysis was performed. At each sampling time, the following 3 variables were recorded: blood venous oxygen saturation (%), venous CO2 and arterial CO2 tension (mmHg), and arterial lactate concentration (mmol/L) from the blood withdrawn. The ΔpCO2 was defined as the difference between the venous partial pressure of CO2 and arterial partial pressure of CO2. The increment (or decrement) rates of these 3 variables were calculated using the following formula: (level of the variable at T1 – level of the variable at T3) / level of the variable at T1. Hemoglobin levels were also recorded at T1, the fifth minute of CPB (T2), and at T3 in all of the patients.

### 2.4. Epidemiologic method and statistical analysis

This prospective observational study met eight methodological items of the MINORS Criteria (Methodological Index for Nonrandomized Studies) [6]. Statistical analysis was performed using IBM SPSS Statistics Version 22 0.0.0 (IBM Corp., Armonk, NY, USA). Quantitative values and scale variables are reported as mean ± SD. Categorical variables were compared by chi-square or Fisher’s exact tests, while continuous variables were compared by Student’s t-test for parametric variables. The Mann–Whitney U test was used for independent variables while paired samples-t and Wilcoxon signed rank tests were used for dependent variables in parametric and nonparametric conditions, respectively. A retrospective power analysis was used to evaluate sample power. It provided a power value of greater than 0.99 in the instance of lactate. Statistical significance was defined at P < 0.05.

## 3. Results

A total of 242 consecutive CABG and heart valve surgery patients met the initial inclusion criteria over a study period of 2 months. Of those patients, 127 had a hemodynamically stable operation period without any major anesthetic or surgical complications and were included in the first analysis. The mean arterial pressures of these patients could be maintained above 65 mmHg before and after CPB, and above 50 mmHg during CPB without any inotropic support except for 5 µg/kg/min dopamine. During CPB, 25 patients were transfused with 1 unit of RBCs for the treatment of dilutional anemia. None of the eligible patients needed 2 units of RBCs (Figure). 

**Figure:**
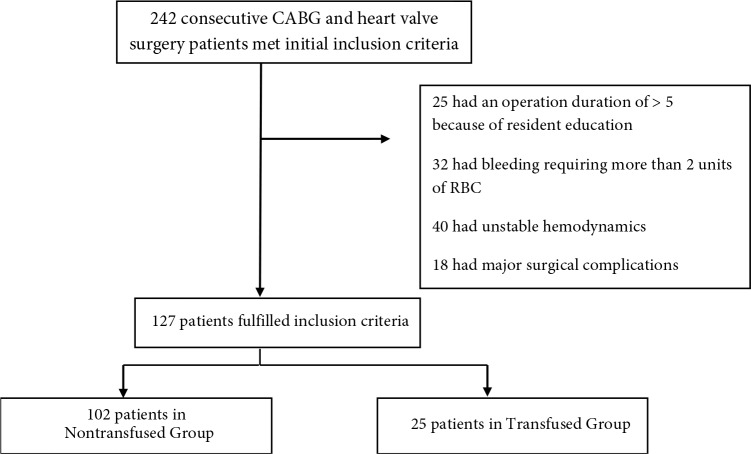
Consort diagram.

Patient characteristics and operative variables are shown in Tables 1 and 2. There was a statistically significant difference in the preoperative hemoglobin values (P = 0.000, P < 0001) between the 2 groups. The patients in Group Tr had lower baseline hemoglobin values, although they were not anemic preoperatively. This group of patients also had significantly lower hemoglobin values after anesthesia induction and at the initiation of CPB. Hemoglobin values did not show any significant difference after the transfusions, at the end of the surgery, between the two groups (Table 3). 

**Table 1:** Patient characteristics.

	Group NTr(n = 102)	Group Tr(n = 25)	P
Sex, female/male	26/76 (25.5/74.5)	9/16 (36.0/64.0)	0.293
Age, years	57.9 ± 9.4	61.04 ± 10.6	0.156
Body surface area, m2	1.81 ± 0.4	1.83 ± 0.5	0.347
Preoperative hemoglobin, g/dL	14.4 ± 1.3	13.1 ± 1.0	0.000
Comorbidities HT	30 (29.4)	7 (28.0)	0.472
DM	10 (9.8)	2 (8.0)	0.164
Preoperative medications Beta-blockers	42 (41.7)	11 (44.0)	0.184
ACEI	14 (13.7)	4 (16.0)	0.234
Diuretics	9 (4.08)	1 (4.0)	0.782
ARA	11 (10.78)	2 (8.0)	0.487
CCB	9 (8.32)	2 (8.0)	0.286
Statin	44 (43.13)	10 (40.0)	0.126
OAD	14 (13.72)	4 (16.0)	0.232

Data are presented as n (%) or mean ± SD. HT: Hypertension, DM: diabetes mellitus, ACEI: angiotensin-converting enzyme inhibitor, ARA: aldosterone receptor antagonist, CCB: calcium channel blocker, OAD: oral antidiabetic agents.

**Table 2:** Operative variables.

	Group NTr (n = 102)	Group Tr(n = 25)	P
Type of operation CABG surgery	82 (80.4)	20 (80)	1.000
Valvular surgery	20 (19.6)	5 (20)	
Operation duration (min)	254.6 ± 45.8	265.8 ± 36.5	0.387
Perfusion duration (min)	92.9 ± 26.0	96.6 ± 20.8	0.606
Cross clamp duration (min)	63.6 ± 23.0	67.8 ± 20.0	0.447

Data are presented as n (%) or the mean ± SD.

**Table 3:** Hemoglobin values measured in both groups (g/dL).

Groups	T1 (before skin incision)	T2 (fifth min of CPB)	T3 (after skin closure)	P (within groups)
Group NTr	13.3 ± 1.5 (13.4; 9.6–16.9)	8.7 ± 1.4(8.6; 7.1–12.9)	9.4 ± 1.1(9.2; 7.8–12.4)	0.000
Group Tr	11.9 ± 1.3(12.1; 9.6–16.3)	6.6 ± 0.3(6.7; 6.1–6.9)	9.0 ± 0.9(8.9; 8.1–11.4)	0.000
P (between groups)	0.000	0.000	0.082	-

Data are presented as the mean ± SD (median; min–max).

There were no significant differences in the ΔpCO2 and ScvO2 levels between the two groups at T1 and T3, respectively. Lactate levels between the groups were also not significantly different at T1, but showed a significant difference at T3. There were statistically significant differences between the ScvO2 and lactate levels at T1 and T3 within both groups and the whole sample. However, the ΔpCO2 levels only changed significantly between T1 and T3 in Group Tr (Table 4).

**Table 4:** Tissue oxidation parameters (ΔpCO2, ScvO2, and lactate) measured in both groups.

	T1 (before skin incision)	T3 (after skin closure)	P (within groups)
ΔpCO2 (mmHg)Group NTr	6.1 ± 2.8 (6.1; 0.1–14.5)	6.1 ± 3.4 (6.0; 0.4–20.3)	0.929
Group Tr	4.9 ± 2.9 (5.2; 0.8–10.1)	8.8 ± 7.1 (7.4; 0.8–31.9)	0.018
Total	5.8 ± 2.8 (5.8; 0.1–14.5)	6.6 ± 4.5 (6.2; 0.4–31.9)	0.264
P (between groups)	0.073	0.057	-
ScvO2 (%)Group NTr	73.2 ± 9.0 (74.5; 45.5–89.8)	64.3 ± 9.6 (63.0; 36.8–85.0)	0.000
Group Tr	71.9 ± 10.1 (71.7; 54.0–88.8)	62.3 ± 9.7 (62.0; 40.4–79.6)	0.003
Total	72.9 ± 9.2 (73.9; 45.5–89.8)	63.9 ± 9.6 (62.7; 36.8–85.0)	0.000
P (between groups)	0.522	0.371	-
Lactate (mmol/L)Group NTr	1.1 ± 0.5 (1.0; 0.2–2.7)	2.2 ± 1.1 (2.0; 0.7–5.6)	0.000
Group Tr	1.0 ± 0.3 (1.0; 0.6–1.8)	2.9 ± 1.6 (2.1; 0.9–7.0)	0.000
Total	1.0 ± 0.4 (1.0; 0.2–2.7)	2.3 ± 1.2 (2.0; 0.7–7.0)	0.000
P (between groups)	0.566	0.009	-

Data are presented as mean ± SD (median; min–max).

The increment rates of ΔpCO2, ScvO2, and lactate levels from T1 to T3 were calculated as described in Section 2.2. There were statistically significant differences in the median increment rates of ΔpCO2 and lactate levels between the NTr and Tr groups (P = 0.015 and P = 0.025, respectively), whereas the difference in the decrement rate of ScvO2 between the NTr and Tr groups did not show any statistical significance (Table 5).

**Table 5:** Increment rates of ΔpCO2, ScvO2, and lactate (T3–T1 /T1).

	Increment rates
ΔpCO2 Group NTr	1.7 ± 9.0 (0.0; –0.9 to 64.0)
Group Tr	2.0 ± 4.3 (0.4; –0.9 to 19.4)
Total	1.8 ± 8.3 (0.1; –0.9 to 64.0)
P	0.015
ScvO2 Group NTr	–0.11 ± 0.17 (–0.13; –0.53 to 0.46)
Group Tr	–0.11 ± 0.18 (–0.06; –0.48 to 0.27)
Total	–0.11 ± 0.17 (–0.12; –0.53 to 0.46)
P	0.591
Lactate Group NTr	1.3 ± 1.2 (0.9; –0.4 to 6.0)
Group Tr	2.3 ± 2.4 (1.6; –0.3 to 10.7)
Total	1.5 ± 1.5 (1.0; –0.4 to 10.7)
P	0.025

Data are presented as mean ± SD (median; min–max).

## 4. Discussion

In this observational prospective study, we explored the global tissue oxygenation parameters among on-pump cardiac surgery patients who had usual surgical periods without any complications except intraoperative dilutional anemia resulting from CPB and who could be treated with one unit of erythrocyte transfusion. The results demonstrated that dilutional anemia resulting from CPB mostly occurs in patients with borderline preoperative hemoglobin concentrations and its correction with RBC transfusion did not normalize the degree of microcirculatory or oxygenation problems, which CPB already makes patients prone to. Patients with dilutional anemia that could be treated with even 1 unit of RBCs were associated with a higher degree of impairment of tissue oxygenation, evidenced by a significant difference in the lactate levels at the end of the operation and incremented rates of ΔpCO2 and lactate, and a nonsignificant difference in the decremented rate of ScvO2**.**

CPB is associated with hemodynamic and metabolic derangements that may influence microcirculatory perfusion and oxygenation [1]. Blood transfusion is frequently performed during cardiac surgery, and ultimately the goal of RBC transfusions is to maintain adequate tissue oxygen delivery by increasing the presence of RBCs at the microcirculatory level [7]. With severe blood loss and large decreases in tissue oxygen concentration, RBC transfusion is a rapid and efficient way to restore the oxygen content [8,9]. However, it can fail to restore tissue oxygen homeostasis in some other clinical settings. 

In particular, studies of patients with sepsis or trauma, as well as patients in medical-surgical intensive care, have reported that RBC transfusion failed to improve microvascular oxygenation [10–14]. The interference between the release of cytokines, vasoregulation, and oxygen consumption in trauma may compromise the efficacy of transfusion [8]. On the other hand, the disappointing effects of RBC transfusion in septic patients may be related to the independence of oxygen consumption and systemic oxygen transport or an inappropriate distribution of oxygen among the tissues. A systematic review by Nielsen et al. also concluded that transfusion did not generally improve microcirculation or tissue oxygenation in intensive care unit patients [15]. 

Yuruk et al. [7] reported that a transfusion of leukodepleted RBCs improved microcirculatory oxygenation and density in a patient with (relatively) healthy microcirculation, as in patients undergoing cardiac surgery with CPB. They assessed sublingual microcirculation via side-stream dark-field imaging and spectrophotometry. Possible explanations for the differences between the results could be the use of global tissue oxygenation markers like ScvO2, ΔpCO2, and lactate and the transfused non-leukodepleted RBCs in the present study. As tissue oxygen consumption is heterogeneous and organ-specific, and the adaptive responses to acute anemia result in heterogeneous organ-specific hypoxic tissue responses, regional monitoring of the microcirculatory functions may not be consistent with the global indices of tissue oxygenation [16]. Furthermore, prestorage leukocyte depletion of blood components reduces the levels of inflammatory mediators and has been shown to improve the outcome significantly in cardiac surgical patients [17,18].

Some studies have revealed that the pretransfusion conditions of the patients were also important determinants of the effects of RBC transfusions on microcirculation. These studies suggested that patients who had microcirculatory disturbances prior to transfusion benefited from the transfusion, showing a significant improvement in tissue oxygenation or microcirculatory flow indices. Conversely, patients who had relatively normal microcirculation showed either no improvement or a decline after transfusion [19]. In the present study, a transfusion of 1 unit of RBCs corrected dilutional anemia resulting from CPB; however, global indices of tissue oxygenation have shown more of a decline in hemodiluted and transfused patients than in patients who did not develop dilutional anemia and were not transfused. It is difficult to determine if this decline arose from the dilutional anemia or transfusion. Karkouti et al. [20] reported that perioperative anemia and RBC transfusion were interrelated risk factors for cardiac surgery-associated acute kidney injury. We think that the higher degree of impairment of tissue oxygenation in the Group Tr patients may also have been interrelatedly affected by dilutional anemia and transfusion. 

The close interplay between RBCs and microcirculatory vessels implements the oxygenation of the microcirculation [7]. A convincing explanation for the different findings of these studies is that the efficacy of transfusion is related to the condition of the microcirculation of the patient, underlying diseases, related medical interventions such as hemodilution or surgical procedures, medications, and the properties of the transfused RBCs [19]. As the ultimate goal of blood transfusion is improving oxygen delivery in states where oxygen consumption is dependent on it, tissue oxygenation and oxygen consumption should be lowered and the host should have a relatively healthy microcirculation prior to transfusion for the improved transfer of oxygen from the blood to the mitochondria; otherwise, the deleterious effects of transfusion become more striking.

Transfusion affects the blood flow, primarily the microcirculation, depending on the transfused RBCs’ deformability, adherence, and aggregability. The modulation of vascular function in opposing ways by activating nitric oxide (NO) production or releasing free hemoglobin, which is a scavenger of NO, is another explanation for the different study findings about the efficacy of transfusions. The presence of white blood cells in the transfused units may also have an important role in the adverse effects observed in patients [7,19]. In order to minimize these various effects, RBCs that had been stored for fewer than 10 days were used in this study.

It is natural that patients with preoperative borderline hemoglobin levels have a higher probability of transfusion because of hemodilution. Although patients with preoperative anemia were excluded from our study, patients who had preoperative borderline hemoglobin values had significantly lower hemoglobin values after anesthesia induction and the initiation of CPB because of hemodilution. Although the hemoglobin levels did not differ significantly at the end of the operation, the tissue oxygenation parameters of the Group Tr patients were impaired. 

This trial had some limitations. First, it involved a small sample of patients, based on which strong recommendations cannot be made. A further limitation was that only the lactate levels at the end of the surgery showed a significant difference between the transfused and nontransfused groups, probably because of the small sample of patients, as the increment rate of ΔpCO2 significantly differed and the decrement rate of ScvO2 nearly significantly differed between the groups. 

In conclusion, we have shown that the dilutional anemia that results from CPB mostly occurs in patients with borderline preoperative hemoglobin concentrations and its correction with 1 unit of RBC transfusion during on-pump cardiac surgery does not normalize, or even increases, the risk of microcirculatory oxygenation problems, which the patients are already prone to because of the nature of CPB. We think that dilutional anemia and transfusion probably have synergistic effects on oxygenation problems in the microcirculation. Preventing these, especially in patients with preoperative borderline hemoglobin levels, may therefore reduce the burden of impaired microcirculation-associated organ failure in on-pump cardiac surgery. Future prospective trials with a large sample of patients must be performed to evaluate physiologic transfusion triggers in combination with existing clinical and laboratory indices of oxygen-supply dependency.
